# Inactivation of Prions by Low-Temperature Sterilization Technology Using Vaporized Gas Derived from a Hydrogen Peroxide–Peracetic Acid Mixture

**DOI:** 10.3390/pathogens10010024

**Published:** 2020-12-31

**Authors:** Akikazu Sakudo, Daiki Anraku, Tomomasa Itarashiki

**Affiliations:** 1 School of Veterinary Medicine, Okayama University of Science, Imabari, Ehime 794-8555, Japan; 2 Laboratory of Biometabolic Chemistry, School of Health Sciences, University of the Ryukyus, Nishihara, Okinawa 903-0215, Japan; 3 Saraya Co., Ltd., Chuo-ku, Osaka 541-0051, Japan; anraku@saraya.com (D.A.); itarashiki@saraya.com (T.I.)

**Keywords:** hydrogen peroxide, peracetic acid, prion, scrapie, sterilization

## Abstract

Prion diseases are proteopathies that cause neurodegenerative disorders in humans and animals. Prion is highly resistant to both chemical and physical inactivation. Here, vaporized gas derived from a hydrogen peroxide–peracetic acid mixture (VHPPA) was evaluated for its ability to inactivate prion using a STERIACE 100 instrument (Saraya Co., Ltd.). Brain homogenates of scrapie (Chandler strain) prion-infected mice were placed on a cover glass, air-dried, sealed in a Tyvek package, and subjected to VHPPA treatment at 50–55 °C using 8% hydrogen peroxide and <10% peracetic acid for 47 min (standard mode, SD) or 30 min (quick mode, QC). Untreated control samples were prepared in the same way but without VHPPA. The resulting samples were treated with proteinase K (PK) to separate PK-resistant prion protein (PrPres), as a marker of the abnormal isoform (PrP^Sc^). Immunoblotting showed that PrPres was reduced by both SD and QC VHPPA treatments. PrPres bands were detected after protein misfolding cyclic amplification of control but not VHPPA-treated samples. In mice injected with prion samples, VHPPA treatment of prion significantly prolonged survival relative to untreated samples, suggesting that it decreases prion infectivity. Taken together, the results show that VHPPA inactivates prions and might be applied to the sterilization of contaminated heat-sensitive medical devices.

## 1. Introduction

Prion diseases, also known as transmissible spongiform encephalopathies (TSEs), are a group of fatal neurodegenerative disorders that affect humans and animals [1,2]. Prion diseases develop when an abnormal isoform of prion protein (PrP^Sc^) starts to accumulate in the brain. The abnormal PrP^Sc^ prion has a higher β-sheet content and is proteinase-K (PK)-resistant as compared with its cellular counterpart (PrP^C^) [3,4]. Therefore, resistance to PK is commonly used as a measure of PrP^Sc^ in both clinical diagnosis and laboratory testing [3].

The conversion of PrP^C^ to its conformationally altered form (PrP^Sc^) is a crucial event in the pathogenesis of prion diseases [3,5]. In general, the normal PrP^C^ protein is thought to interact with the abnormal PrP^Sc^ template, resulting in PrP^C^ refolding as PrP^Sc^ [6]. This initial conversion instigates a chain reaction, in which each newly refolded PrP^Sc^ molecule interacts with, and converts, another PrP^C^ molecule, leading to a gradual accumulation of PrP^Sc^ [2,7,8]. The in vitro technique of protein misfolding cyclic amplification (PMCA) resembles this reaction and has been used to examine the amplification potential of PrP^Sc^ in vitro [9,10,11].

Notably, prion agents are recognized to be one of the most resistant pathogens to either physical or chemical inactivation [12]. For example, they are not inactivated by standard sterilization techniques, such as 20 min of autoclaving at 121 °C or ultraviolet/γ-ray irradiation [13,14]. The inactivation of prions requires more severe conditions, for example 18 min of autoclaving at 134 °C [15] or sodium hydroxide and sodium dodecyl sulfate (SDS) treatment—a procedure that is generally considered to be impractical [16]. Furthermore, only a limited number of methods for inactivating prions attached to heat-sensitive medical devices as well as their surface materials have been developed [14,17,18,19].

Vaporized hydrogen peroxide (VHP) (30%–59%) can be used for the low-temperature sterilization of medical devices [17,20]. Several studies have evaluated the inactivation effect of VHP using various VHP sterilizers against a range of pathogens [21,22,23,24,25] including prions [13,26,27,28,29]. Recently, the synergic effect of hydrogen peroxide and peracetic acid on bactericidal and sporicidal activity has also been reported [29,30,31]. The addition of peracetic acid to hydrogen peroxide has advantages in reducing the concentration of toxic hydrogen peroxide for the inactivation, enabling safe and environmentally friendly sterilization methods [20]. As far as we know, however, no studies have evaluated the effect of a vaporized hydrogen peroxide–peracetic acid (VHPPA) mixture on prions. Therefore, it is of interest to determine whether VHPPA mixture has an inactivating effect on prions.

This background prompted us to further investigate the ability of VHPPA to inactivate prions by using the VHPPA instrument STERIACE 100 (Saraya Co., Ltd., Osaka, Japan). We carried out immunoblotting and PMCA of prion, in addition to a mouse bioassay, in order to determine whether VHPPA treatment can decrease the infectivity and proliferation ability of prions.

## 2. Results

**Figure 1:**
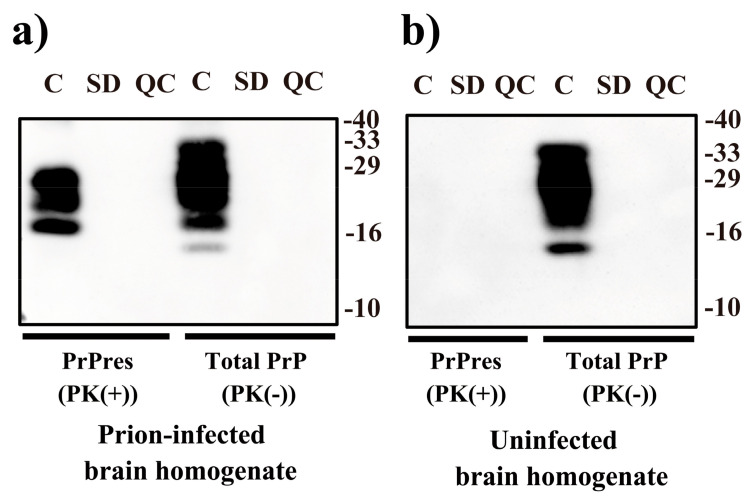
Immunoblot analysis of scrapie prion after treatment with vaporized hydrogen peroxide-peracetic acid (VHPPA). Brain homogenates from prion (Chandler scrapie)-infected mice (**a**) or uninfected mice (**b**) were treated with VHPPA via a STERIACE 100 instrument operating in Standard mode (SD) or Quick mode (QC); an untreated control sample (C) was also prepared. Samples recovered from treatment spots were incubated with proteinase K (PK) (20 μg/mL, 37 °C, 60 min) to estimate PrPres as a marker of PrP^Sc^ (PK(+)) or left untreated to estimate total prion protein (PrP) (PrP^Sc^ + PrP^C^) (PK(−)), and separated by SDS (sodium dodecyl sulfate)-polyacrylamide gel electrophoresis (PAGE), followed by immunoblotting with SAF83 (anti-PrP) antibody. Molecular weight markers (kDa) are indicated on the right.

To evaluate the effect of VHPPA on prions, brain homogenates from prion (Chandler)-infected mice were collected and subjected to VHPPA treatment. In the present study, we used a mouse scrapie prion (Chandler strain) as a model system, although it should be noted that human prions would be better for to assessing the decrease in infectivity caused by inactivation treatment in the case of studies on the iatrogenic transmission of prions. The VHPPA-treated and untreated samples were examined by immunoblotting using SAF83, an anti-prion protein (PrP) antibody, before PK treatment (PK(−)) and after PK treatment (PK(+)) (Figure 1). Note that the untreated control samples were prepared in exactly the same way as the VHPPA-treated samples except that VHPPA was not applied. PK-resistant PrP (PrPres) was used as an index of PrP^Sc^, while total PrP measured before PK treatment included both PrP^Sc^ and PrP^C^. As compared with the untreated control sample, the intensities of PrPres and total PrP were lower in samples exposed to Standard mode (SD) and Quick mode (QC) VHPPA treatment. These results suggest that both PrP^Sc^ and PrP^C^ were degraded by VHPPA treatment under both SD and QC conditions.

**Figure 2:**
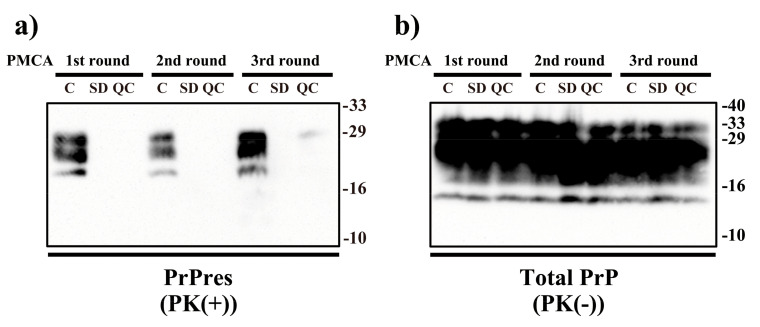
Inhibition of protein misfolding cyclic amplification (PMCA) by VHPPA treatment of scrapie prion. In PMCA, normal mouse brain homogenate was used as the PrP^C^ substrate. Untreated control sample (C) or VHPPA-treated samples under SD and QC conditions were applied to 3 rounds of PMCA. After each round, aliquots were treated with PK (20 μg/mL, 37 °C, 60 min) (PK(+)) (**a**) and untreated (PK(−)) (**b**) and then separated by SDS-PAGE, followed by immunoblotting with SAF83 (anti-PrP) antibody. Molecular weight markers (kDa) are indicated on the right.

Next, we investigated the in vitro proliferation ability of PrP^Sc^ after VHPPA treatment by PMCA using brain homogenate from uninfected C57BL6/J mice as the PrP^C^ substrate (Figure 2). The untreated and VHPPA-treated samples were diluted 10-fold with PrP^C^ substrate and a first round of PMCA amplification was carried out. The resulting samples were again diluted 10-fold with PrP^C^ substrate and a second round of PMCA amplification was carried out; this procedure was repeated for a third round of PMCA. After each PMCA round, an aliquot was removed and treated with PK (PK(+)) or without PK (PK(−)), followed by immunoblotting with SAF83 (anti-PrP) antibody. As a result, the PrPres band was detected after all three rounds of PMCA for the untreated control sample, suggesting that PrP^Sc^ in the untreated control sample was amplified by PMCA. By contrast, no PrPres band was observed in any of the three PMCA rounds for the both the SD and QC VHPPA-treated samples. On the other hand, bands of total PrP, mainly corresponding to the PrP^C^ substrate, were similarly detected in all PK(−) samples after all three rounds of PMCA. Overall, our findings indicate that the ability of prion to proliferate in vitro was decreased by the 2 VHPPA treatments. Taken together, these observations suggest that both SD and QC VHPPA treatment eliminates the amplification ability of PrP^Sc^.

**Figure 3:**
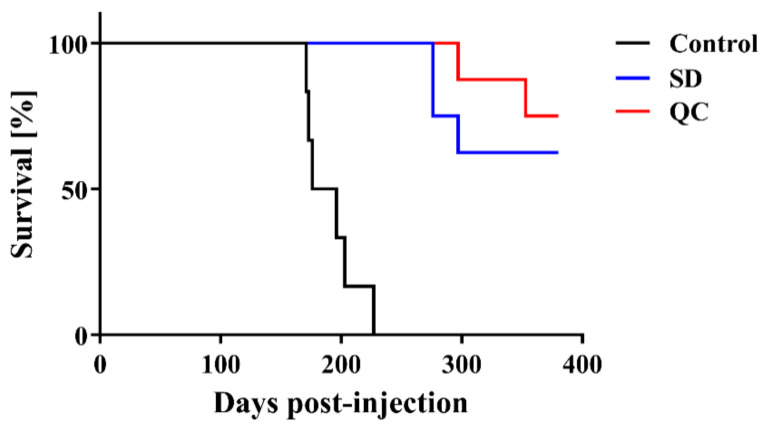
Survival of mice infected with scrapie prion that had been treated with VHPPA. Mice were injected intracerebrally with either VHPPA-treated brain homogenate from Chandler prion-infected mice (blue line, SD; red line, QC) or untreated brain homogenate from infected mice (black line, Control). The survival curves were compared by log-rank test with *p* < 0.01 considered statistically significant.

Lastly, we intracerebrally injected C57BL/6J mice with VHPPA-treated (SD and QC) or untreated brain homogenate obtained from Chandler prion-infected mice to determine infectivity in vivo. The three groups were compared for survival and disease incubation time. Comparing the survival curve among the three groups (Figure 3), all six mice injected with untreated brain homogenate showed ataxia and tremors before 227 days, whereas none of the mice injected with VHPPA-treated (SD or QC) prion died before 275 days. The survival curve differed between the control group and the SD or QC treatment groups (log-rank test, both *p* < 0.01), while there was no significant difference between SD and QC.

**Figure 4:**
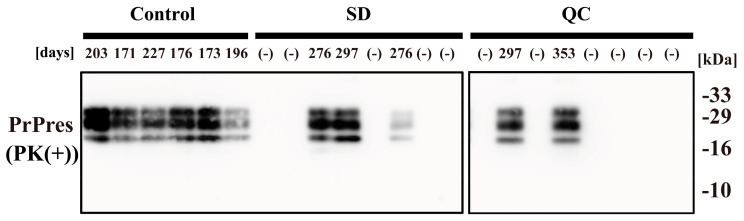
Immunoblot analysis for PrPres in the brain of mice that died. Mice were injected intracerebrally with brain homogenate from Chandler prion-infected mice that had been treated with VHPPA (SD and QC conditions) or with untreated brain homogenate from infected mice. The mice were euthanized either at end-stage disease when they displayed ataxia and tremors (the timepoint is indicated in days) or at 381 days if they were disease-free (–). Brain samples were collected, treated with PK (PK(+)), and then analyzed by SDS-PAGE followed by immunoblotting with SAF83 (anti-PrP) antibody. Molecular weight markers (kDa) are indicated on the right.

Among the three groups, the disease incubation time reflected a similar tendency as the survival curve (Table 1). Mice injected with untreated brain homogenate had a shorter mean disease incubation time (191.0 ± 9.0 days) as compared with mice injected with VHPPA-treated brain homogenate (SD group, >276 days; QC group, >297 days). Furthermore, immunoblot analysis showed that PrPres was present in the brain of all mice that died, including all six control mice, three in the SD group, and two in the QC group (Figure 4), confirming that the cause of death in these mice was prion disease. Collectively, our findings indicate that, in a mouse model of prion infection, VHPPA treatment (SD or QC mode) of scrapie prion slows down the onset of disease symptoms and increases survival.

## 3. Discussion

Recently, the use of oxidizing agents for the disinfection/sterilization of medical devices has been increasing. Previous studies have shown that a combination of peracetic acid with hydrogen peroxide has a synergistic effect on bactericidal and sporicidal activity and enhances the inactivation efficiency of the individual agents [29,30,31]. In addition, peracetic acid is known to attack germination receptor proteins located in or at the spore inner membrane of microorganisms [32], while hydrogen peroxide increases the penetration of peracetic acid by compromising the microorganism’s spore coat, which provides the main resistance against the combined agents. Thus, the synergy may occur between two oxidizing agents that attack different sites within the target organism [33].

To date, there have been no studies investigating the effect of VHPPA on prion inactivation. On the one hand, two studies have shown that peracetic acid (1500 ppm, room temperature, 20 min; or 0.2%, room temperature, 1 h) has an inactivation effect on prions [15,34]. On the other hand, some guidelines state that peracetic acid is ineffective or sub-optional for decontamination and reducing the infectivity of prions [18,35]. By contrast, a few studies have shown that VHP inactivates prions [15,27,28]. For example, it is well established that hydrogen peroxide gas plasma can inactivate prions [36]; with this technology, hydrogen peroxide gas is used for the sterilization process, while the plasma is generated at a later stage of the cycle and is not thought to play a significant, if any, role in sterilization [37,38].

Interestingly, liquid hydrogen peroxide has no effect on prions. It has been shown that VHP increases sensitivity to proteases by causing unfolding and fragmentation of proteins; by contrast, liquid hydrogen peroxide inhibits proteolytic digestion because it causes protein aggregation, which inhibits proteolytic digestion [27]. Applied under vacuum conditions, VHP may show enhanced penetration into proteins, further increasing prion inactivation [27]. The present finding of prion inactivation by using a STERIACE 100 instrument under depressurization with the injection of a hydrogen peroxide–peracetic acid mixture at very low concentrations (hydrogen peroxide, 8%; peracetic acid, <10%) is consistent with the above notions of (1) enhanced penetration of oxidizing agents under vacuum conditions and (2) increased inactivation efficiency due to a synergic effect between the two reagents.

There are a variety of other potential methods for inactivating prions on heat-sensitive medical devices, including VHP [28], hydrogen peroxide gas plasma [15], copper (0.5 mmol/L) plus hydrogen peroxide (100 mmol/L) (1 h, room temperature) [15], and electrically charged disinfectants [39]. Future work should assess the effectiveness of these approaches relative to VHPPA treatment, in addition to combinations of treatments. Further studies on this issue will lead to the development of more effective disinfection/sterilization procedures.

In the present study, there was a clear difference between the PMCA and in vivo results, which were not totally concordant. It appears that after three consecutive rounds of PMCA, prion could not be detected in the VHPPA-treated samples. However, when these samples were inoculated in vivo, there were animals that succumbed to disease. This may be due to PrP^Sc^ quantity and implies that the sensitivity of the mouse bioassay is superior to that of PMCA.

The present study used intracerebral inoculation with 10% brain homogenate, which is considered to be an excessive dose relative to other studies using scrapie prion-contaminated wires [40]. Another report also suggests that the 10% brain homogenate inoculum (protein content, ~2000 µg) may be overloaded because protein contamination on surgical instruments after use generally ranges from 8 to 91 µg per instrument [41]. However, it should be noted that the present study confirmed the inactivation effect on prions by VHPPA under even more severe conditions using intracerebral inoculation with 10% brain homogenate.

A recent study of PMSA (Protein Misfolding Shaking Amplification) using glass, Teflon^®^, zirconia/silica, and stainless steel has shown that the effect of decontamination treatment is highly dependent on the treated surface material [19]; thus, further studies on the effect of VHPPA on prions using various surface materials would be interesting.

The study has a few limitations. First, no quantitative results were obtained in the mouse bioassay. Further studies using a quantitative approach based on, for example, endpoint titration [42] or incubation time interval assay [43], are needed to quantify the reduction in infectivity achieved by VHPPA. Secondly, we did not optimize the conditions of the PMCA and the assay was not quantitative. In the future, the ratio of PrPres to PrP^C^ in the PMCA reaction should be varied to achieve more quantitative data. Alternatively, experiments using quantitative real-time quaking-induced conversion (qRT-QuIC) should be performed. Thirdly, in the present study, simulated soiling of prion samples with, for example, organic matter was not performed; furthermore, only one type of sample, mouse brain homogenate, was subjected to VHPPA treatment. To mimic the conditions of used medical devices, the effects of VHPPA treatment on prions in different source materials (i.e., tissue or cell samples) should be determined. Lastly, the study did not compare VHPPA with other conventional prion inactivation methods. Further comparative studies should be performed to identify effective treatments with data on the relative inactivation efficiency of different methods, as well as potential improvements due to a combination of methods.

Prion diseases are considered to be a prototype of neurodegenerative protein misfolding diseases [44,45]. This hypothesis is supported by the evidence that other neurodegenerative protein folding diseases seem to involve a similar mechanism, in which self-perpetuating protein aggregation occurs [45,46]. This type of prion-like transmissibility is seen in Alzheimer’s disease (AD) and Parkinson’s disease (PD) [47]. The present study demonstrates that VHPPA significantly reduces the proliferation of scrapie prion, suggesting that it might also be used to treat medical devices contaminated with aggregated products of other neurodegenerative diseases such as AD and PD. Further studies will be needed to determine whether VHPPA effectively inactivates these other aggregation-prone proteins.

## 4. Materials and Methods

### 4.1. VHPPA Treatment of Prions

A 20-µL aliquot of 10% (w/v) of prion (mouse-adapted scrapie, Chandler strain)-infected mouse brain homogenate in phosphate-buffered saline (PBS) (Life Technologies, Carlsbad, CA, USA) was spotted onto a cover glass and air-dried. The cover glass (18 × 32 mm thickness No.1, 0.12–0.17 mm, Matsunami Glass Ind., Ltd., Osaka, Japan) was placed in a Tyvek bag (Sterilization bag 300 mm, 45284, Saraya Co., Ltd., Osaka, Japan) and processed with the VHPPA sterilizer instrument STERIACE 100 [48]. After treatment, each resultant sample spot was collected with 200 μL of PBS.

The STERIACE 100 instrument performs 3 processes: (1) initial depressurization, where the chamber is depressurized from atmospheric pressure to 59 Pa; (2) sterilization, consisting of a further depressurization step, a hydrogen peroxide–peracetic acid injection step, and a sterilization step; and (3) ventilation. There are 2 modes of sterilization process: standard mode (SD) and quick mode (QC), both of which use VHPPA derived from non-concentrated 8% hydrogen peroxide and <10% peracetic acid [20]. One cycle of STERIACE 100 treatment includes 2 sterilization processes at 50–55 °C, followed by 4 ventilation processes. The total process time is 47 min for SD and 30 min for QC.

### 4.2. Prion Injection

To evaluate the change in infectivity of prions before (Control) and after VHPPA treatment (SD and QC), each sample recovered from each of the spots on the cover glass were intracerebrally injected into C57BL/6J mice. Via a microsyringe, 20 µL of the recovered sample was injected into the ventricular system in the brain of each mouse. To calculate the disease incubation time, the mice were observed daily for clinical symptoms such as tremors and ataxia. 

### 4.3. Immunoblot Analysis

The protein concentration in each sample recovered from each of the spots before and after VHPPA treatment was measured by a Bio-Rad DC protein assay kit (Bio-Rad, Hercules, CA, USA). Samples (50 μL containing 100 μg of protein) were incubated with PK (20 μg/mL) at 37 °C for 60 min to discriminate PrPres. Next, 50 μL of 2× SDS gel-loading buffer (90 mM Tris/HCl (pH 6.8), 2% SDS, 10% mercaptoethanol, 20% glycerol, and 0.02% bromophenol blue) was then added and the samples were boiled at 100°C for 10 min to stop the reaction. Samples treated in the same way except for PK digestion were also prepared. We separated the proteins by SDS-PAGE (15%) as described previously [39] and transferred them to polyvinylidene difluoride (PVDF) membranes (Amersham Biosciences, Piscataway, NJ, USA) by means of a semidry blotting system (Bio Rad, Cambridge, MA, USA). The membranes were blocked by incubation with 5% skim milk (Wako, Osaka, Japan) at 37 °C for 1 h, and then incubated with SAF83 (anti-PrP) antibody (SPI bio, Montigny le Bretonneux, France) in PBS-T (PBS containing 0.1% Tween 20) containing 0.5% skim milk for 1 h at 37 °C. The membranes were subsequently washed 3 times in PBS-T for 10 min each, before being incubated with secondary antibody (horseradish peroxidase-labeled anti-mouse immunoglobulin; Jackson Immunoresearch, West Grove, PA, USA) in PBS-T containing 0.5% skim milk at 37 °C for 1 h. After 3 washes with PBS-T for 10 min each, the blots were developed with ECL reagent (Amersham Bioscience, Piscataway, NJ, USA), and chemiluminescence was measured by Ez-Capture MG (ATTO Corp., Tokyo, Japan).

### 4.4. PMCA

To carry out PMCA, we used an automatic cross-ultrasonic protein-activating instrument (ELESTEIN 070-GOT, Elekon Science Corp., Chiba, Japan) [49]. Each samples derived from spots treated with VHPPA (SD and QC) or untreated (Control) were sealed in a capped polystyrene tube (catalogue number 035-12, Elekon Science Corp.) and subjected to 40 cycles of amplification, each comprising sonication and then 1 h of incubation at 37 °C. The samples were gently agitated throughout the process. After each round of amplification, the product was diluted 1:10 with PrP^C^ substrate [10% C57/BL6J mouse brain homogenate in PBS containing 4 mM EDTA, 1% Triton X-100, and complete protease inhibitors (Roche Diagnostics, Mannheim, Germany)], and applied to another round of PMCA. This process was repeated until 3 rounds had been carried out. After PMCA and PK treatment, the samples were subjected to immunoblotting using SAF83 antibody as described above.

### 4.5. Statistical Analysis

PMCA Survival curves were analyzed by log-rank test using GraphPad Prism 7 software (GraphPad Prism Software Inc., La Jolla, CA, USA).

## 5. Conclusions

In this study, the effect of VHPPA on prion inactivation was evaluated at both the in vitro and in vivo level. When scrapie prions were treated with VHPPA under standard and quick conditions using a STERIACE 100 instrument, prions were significantly degraded, and both in vitro proliferation and in vivo prion infectivity were reduced. These findings suggest that VHPPA is effective for prion inactivation and may be used for sterilization of prion-contaminated medical devices.

## Figures and Tables

**Table 1:** Disease incubation time among mice injected with VHPPA-treated scrapie prion.

Treatment	Mean Incubation Time ± SEM ^1^	*N*/*N_0_*^2^
Control	191.0 ± 9.0 days	6/6
SD	>276 days ^3^	3/8
QC	>297 days ^4^	2/8

^1^ SEM, standard error of the mean. ^2^ *N*, number of dead animals; *N_0_*, number of inoculated animals. ^3^ Death of 2 mice at 276 days and 1 mouse at 297 days, while 5 mice survived more than 380 days. ^4^ Death of 1 mice at 297 days and 1 mouse at 353 days, while 6 mice survived more than 380 days.

## Data Availability

The prions protein mentioned in this study can be retrieved in NCBI.
